# HIV Risk Perception and Behavior among Sex Workers in Three Major Urban Centers of Mozambique

**DOI:** 10.1371/journal.pone.0094838

**Published:** 2014-04-15

**Authors:** Judite Langa, César Sousa, Mohsin Sidat, Karen Kroeger, Eleanor McLellan-Lemal, Hrishikesh Belani, Shama Patel, Daniel Shodell, Michael Shodell, Irene Benech, Richard Needle

**Affiliations:** 1 Centers for Disease Control and Prevention, Maputo, Mozambique; 2 Faculty of Medicine, Eduardo Mondlane University, Maputo, Mozambique; 3 Centers for Disease Control and Prevention, Atlanta, Georgia, United States of America; 4 Long Island University, New York, New York, United States of America; 5 Office of the Global Aids Coordinator, Washington, District of Columbia, United States of America; International AIDS Vaccine Initiative, United States of America

## Abstract

HIV risk perceptions and behaviors of 236 commercial sex workers from three major Mozambican urban centers were studied using the International Rapid Assessment, Response and Evaluation (I-RARE) methodology. All were offered HIV testing and, in Maputo, syphilis testing was offered as well. Sixty-three of the 236 opted for HIV testing, with 30 (48%) testing positive for HIV. In Maputo, all 30 receiving HIV tests also had syphilis testing, with 6 (20%) found to be positive. Results include interview excerpts and qualitative results using I-RARE methodology and AnSWR-assisted analyses of the interviews and focus group sessions.

## Introduction

With a national Human Immunodeficiency Virus (HIV) prevalence of 11.5% among individuals aged 15–49 years, Mozambique is one of the countries most affected by HIV/Acquired Immunodeficiency Syndrome (AIDS) [1]. HIV prevalence in Mozambique is higher among women than men (13.1% vs. 9.2% respectively) and higher in urban than rural areas (15.9% vs. 9.2% respectively) [1]. The sheer magnitude of implementing prevention interventions and treatment among the general Mozambican population has made it difficult to focus on marginalized populations that may be at significantly higher risk of HIV infection, such as female commercial sex workers (CSW).

Mozambique lacks comprehensive data on the extent of commercial sex work and its importance in the HIV epidemic. The mean age of female sexual debut in Mozambique is 16.5, however 23.1% of females in the 15–19 age group and 26.7% of those in the 20–24 age group had sexual debut before the age of 15 years [1]. Early sexual debut is an established determinant for acquiring of HIV and other sexually transmitted infections (STI) [2], [3]. The mean age for women first entering sex work in Mozambique is 17.8, with a range of 9–28 years [4]. In Mozambique there are no laws, legislation, or regulations that pertain to sex work, and CSW is not prohibited. However, female CSW still face stigma and discrimination, often do not have provision of health services tailored to their needs, and have difficulties accessing regular services provided to the general population [5]. Given the greatly increased risk of HIV exposure attendant to CSW, it is important to have information on the behaviors of CSW, their perceptions of risk, and their outcomes to inform the creation and implementation of successful AIDS health and prevention measures.

In order to obtain this information on CSW behaviors and perceptions, we carried out interviews and focus groups with CSW, their male clients, drug users, service providers and policy makers in Mozambique's three largest port cities: Nacala in the north; the central port of Beira; and the capital, Maputo, located in the south of the country. In this paper, we report results from the interviews with CSW. Separate papers on the male CSW clients, drug users, service providers and policy makers who were active study subjects are forthcoming.

## Methods

Between November 2007 and January 2008, we interviewed 236 CSW, 47 male clients of CSW, 13 policy makers and 50 health service providers. Table 1 below shows the CSW interviewed study site and type of interview. Details of other interviewed groups will be presented in the respective forthcoming papers.

**Table 1:** Commercial sex-worker interviews and focus groups by study site, Mozambique, 2007–2008.

Number of participants in Key interviews (KI) and focus groups (FG)						
Nacala		Beira		Maputo		Total
KI	FG	KI	FG	KI	FG	
43	42 (6)*	38	28 (4)*	48	37 (7)*	236

*Numbers in parentheses = numbers of focus groups conducted.

### Data Collection

Qualitative data collection was conducted using the I-RARE methodology. Based on established anthropological techniques, I-RARE was developed and refined to gather public health information, and is especially useful for gathering data on populations that are hard to reach and about which little is known [6]–[8]. Often these are marginalized segments of society, such as the CSW comprising this present study. The method employs a suite of complementary qualitative data collection methods, including in-depth semi-structured interviews and focus group discussions conducted by trained members of the community with extensive knowledge about the issues under investigation, to explore and better understand the context in which risk behaviors take place. Per established I-RARE methodology, the approach is designed to generate rich, in-depth descriptive information, and necessarily relies on small samples which may not be statistically representative of the population. Sampling was conducted for variables of interest, namely residence in the community, and participation in certain risk behaviors (sex work, drug use). I-RARE methodology has been used in South Africa [8]–[11] and Cambodia [12] to better understand drug use and sexual practices that place injection and non-injection drug users (IDUs/NIDUs), commercial sex workers (CSWs) and Men who have Sex with Men (MSM) at risk for HIV. The assessments elicited important information that guided programmatic recommendations for designing specific target interventions for these key populations.

The interview and focus group guides used in the present study are included as supplemental material (S1 and S2, respectively). Each interview was conducted by two trained staff, one conducting and recording the interview and the other taking notes. Study sites were selected based on proximity to ports or transport corridors likely to generate sex work, and presence of areas or neighborhoods identified by local experts as locations where sex work, drug trafficking, and drug use activities are taking place.

In each of the three study sites, existing local organizations already working with CSW were identified and recruited for participation in the data collection phase. Individuals from these organizations – often themselves former CSW or others actively involved in implementing HIV-related activities with this population – were trained to conduct field observations, ethnographic mapping, in-depth key informant interviews and focus group discussions. Each of the three study sites had a field team of 10–15 members that worked in pairs of two, with linkages to accredited voluntary HIV counseling and testing (VCT) team member seconded from organizations providing routine VCT services.

In each city preliminary mapping exercises were undertaken to first identify “hotspots” – locations where CSW was taking place. A determination was then made of optimal locations within the hotspot, and the days of the week and times of day when activity was greatest. Once a group of CSW was identified and enrolled – with the criteria that they be at least 18 years of age, not exhibit violent behavior or signs of mental illness at the time of the enrollment, acknowledge having exchanged sex for money or other items (e.g., food, clothing, gifts) within the past 30 days, and working in an identified high-risk venue – they were asked to help recruit others who they might know as part of a snowball sampling approach for expanding the study to other potential participants. In accordance with Mozambican Ministry of Health guidelines, no monetary payments or incentives were made or offered to assessment participants. Free VCT was offered and, where accepted, performed according to Mozambique national guidelines using a serial two-test algorithm employing Determine and Unigold rapid tests.

Analysis included data from all sources, including mapping and observational data, key informant and focus group interviews, and socio-demographic survey data. Triangulation, a method of comparing results across data sources, data collection methods, and sites, were used to validate the findings. We attempted to reduce social desirability bias by training field teams and interviewers to be non-judgmental and professional in their dealings with participants, and by working closely with local organizations that have established relationships in the community.

Confidentiality safeguards were maintained at all stages and participants offered VCT for HIV and testing positive were referred for HIV care and treatment to health facilities as per National Guidelines. Ethical approval of the study was obtained through the Mozambique Ministry of Health IRB and the United States Centers for Disease Control and Prevention in July 2007. All participants read and signed informed consent prior to participating in the study.

### Data analysis

Qualitative data analysis was conducted in three phases. First, the field teams debriefed together on a daily basis to identify emerging concepts including beliefs, values, attitudes, knowledge, or practices described by participants in response to open-ended questions within a given topic area or domain of inquiry. Recorded interviews were transcribed and imported into AnSWR, a computer-assisted software for qualitative data analysis [13]. Second, an analysis team developed a codebook and with the AnSWR support identified emerging ideas, labeled relevant text and coded the transcribed interviews. A summary with main and recurrent themes was developed. Third, analyses were conducted across data sources and sites to identify common patterns and salient themes which were compiled with the assistance of *Word for Windows*.

Socio-demographic, HIV and syphilis testing data were analyzed using SPSS version 15.0 for Windows. A coding system was used and no names were collected for any of these data.

## Results

### Socio demographic characteristics of study participants

The characteristics of the 236 CSW from the three centers are summarized in Table 2. Age distribution and education levels of the CSW were similar for the three cities and religious affiliations follow the same pattern as the general population (south and center regions mainly Christians, and the north mainly Muslims). More CSW in Beira had children than in the other cities, and Beira respondents also were more likely to respond that they regarded as their main occupation as something other than sex work. CSW in Maputo were more likely to state their main occupation as sex workers than participants from Beira and Nacala.

**Table 2:** Characteristics of all 236 Commercial Sex Workers Interviewed, Mozambique, 2007–2008.

		Maputo City (n = 85)	Beira (n = 66)	Nacala Porto (n = 85)
Age of the participants (in years)	Average	23	25	25
	Median	22	23	23
	Range	18–40	18–42	18–48
Education level	None	0% (0/83)	4% (2/46)	3% (2/76)
	Primary	66% (55/83)	57% (26/46)	58% (44/76)
	Secondary	34% (28/83)	39% (18/46)	39% (30/76)
Religion	Christian	78% (61/78)	70% (44/63)	37% (31/84)
	Muslim	1% (1/78)	20% (13/63)	49% (41/84)
	Traditional	9% (7/78)	5% (3/63)	6% (5/84)
	None	12% (9/78)	5% (3/63)	8% (7/84)
Have children*	Yes	52% (34/65)	82%(46/56)	62%(48/78)
	No	48% (31/65)	18%(10/56)	38%(30/78)
	Live with children **	97% (33/34)	94% (43/46)	81% (39/48)
	No response	23% (20/85)	15%(10/66)	8%(7/85)
Have regular partner*	Yes	60% (36/60)	49% (17/35)	32% (19/59)
	No	40% (24/60)	51% (18/35)	68% (40/59)
	Live with partner **	31% (11/36)	59% (10/17)	47% (9/19)
	No response	29% (25/85)	47% (31/66)	31% (26/85)
Main Occupation*	Sex work	62% (51/82)	26% (14/53)	47% (40/85)
	Student	10% (8/82)	23% (12/53)	27% (23/85)
	Domestic	5% (4/82)	2% (1/53)	6% (5/85)
	Street vendor	2% (2/82)	15% (8/53)	9% (8/85)
	Bar worker	0	0	6% (5/85)
	Other	21% (17/82)	34% (18/53)	5% (4/85)
	No response	4% (3/85)	20% (13/66)	0
				

*Calculations based only on number actually responding to the question.

**Calculations based only on those responding ‘yes’ to the question.

### Initiation and Motivations for entering commercial sex work

The two main reasons for entering CSW given in this study were either or obtaining basic subsistence necessities for themselves and their families, or in order to obtaining perceived desirable items – fashionable clothes, shoes, higher-end consumer items – which would otherwise have been unaffordable:

*What led me to do this, to have sex in exchange for money were my living condition. I have nothing, I do not work, I have children to raise, so, to be able to eat I depend on this sex work money. (Age 33, Nacala)*

*[It was] because of my friends, as they were always dressed up to go to school. I also started doing this work to get money to buy similar clothes. Since my father was not working, my mother does not work, so I had no money to be gorgeous like my friends. (Age 27, Nacala).*

The desire to be financially independent and support themselves away from their parents' homes was an additional common theme. Thus, while the motivations may differ, the fundamental driving force for the work described was financial gain.

### Organization of commercial sex work: strategies for recruiting clients, earnings

The sex workers in this study were autonomous and not working for someone else, nor were involved in any type of CSW organization or agency (these may exist in the three cities – however only street-based CSW were recruited in the present study), they did sometimes enlist the help of other persons who acted as a broker or middleman. This could also involve a small cost to the workers. Such persons directed clients to the CSW, being in a better position to approach potential clients. This could involve such things as the ability to speak English and thus being able to recruit clients who are foreigners (tourists, sailors, and foreign workers residing in the country). Some participants reported paying the middleman a small fee for their services, for example 50–200 meticais (about $2–$8 USD), though none of the participants described the arrangements with middlemen as formal:

*There is a kid, who is the son of my neighbor. Sometimes he goes into the harbor. As he can speak English he goes there to speak with them [potential clients]. They come out and he brings them here, to my house or to the bar where I am sitting. Then I take some fifty meticais [around 2 US dollars] and give to the kid … by way of gratitude. (Age 33, Nacala)*

*There are the hotel staff that may help. When the white clients arrive, the hotel staff call us. When we get paid 1000 [meticais] we take some of it to give them 200 [meticais]. (Age 21, Maputo)*

CSW from Maputo reportedly earned more than those in Beira and Nacala, with earnings in Beira being the lowest. Participants reported generally charging from 50 meticais (about $2 USD) to 500 meticais (about $20 USD) per sex act. Across the cities, participants reported a range of one to 10 clients per day, with an average of three to four per day. Weekends (Friday through Sunday) were uniformly reported as the highest volume days, with evening hours the busiest times. Mondays and Wednesdays are also reported as days that could be busy. Some participants reported only working two to four days per week.

Clients were foreign as well as Mozambican. They also varied by occupation, and included truck drivers, sailors or ship crew officials, and tourists from different countries, but no specific patterns were found regarding country of origin of foreign clients. Many participants stated a preference for foreign clients because of better payment for their services. On the other hand, while a transaction with a local client may occur in the client's car and last a relatively short time, usually one hour, a transaction with a foreign client might occur in the client's hotel room and could last for the whole night:

*A number of them [clients] are workers, but I don't know where they coming from. Some say they are from the railways. Others are self-employed. Others are industrial workers. But asking where they come from also means you want some kind of engagement. I did not want any engagement, hence I was not interested knowing who is and where he comes from. (Age 45, Beira)*

*If I am with a local man, he never takes longer than one hour of time. Those foreigners are already mine. They call me and I go to the hotel. I go there at 11p.m. and may stay there until sunrise. In the morning, I wake up and go back home. When clients are from here it is a matter of 45 minutes to 1 hour of time and afterwards they leave. (Age 32, Nacala)*

### Sexual practices

In general, CSW reported that they would not agree to have oral or anal sex with a client and would forego the income from that kind of sexual transaction but others reported engaging in anal sex for higher prices. Anal sex was considered by some participants as potentially a great health risk but it was not clear whether it occurred with condoms and/or lubricants.

*There are others clients that like [oral sex]. That is such a strange type of sex! For me it's only the vagina. Ass, no, not even a man that may give me a ring (husband). No, I cannot. Even if it means going home empty handed. (Age 24, Maputo)*

*Only those whites like doing anal sex and I accept and they pay me a lot of money. It only is a pity that I… Find some time for me to show you how my ass is now. I am totally messed up because of the anal sex I had with my clients. (Age 33, Nacala)*

### Knowledge about condoms and condom use

All participants knew what a male condom was, and most understood the protective effects of the male condom against STIs, HIV, and pregnancy. With regards to its usage, some CSW reported always requesting condom use during sex with clients, and even refusing clients who did not use condoms.

*I always force my clients to use a condom. I have never accepted having sex without a condom. Even if a client is a regular, we have to use a condom. It is not the case of saying I got familiar and acquainted with him and there is no problem, we don't have to use a condom. No, it is always with condom, yes. If the client does not agree with the conditions then very sorry there will be nothing. (Age 42, Beira)*

Other participants, however, reported that they engaged in unprotected sex with clients who did not want to use a condom, usually charging a higher price in these circumstances.

*If the client wants to have a sexual relation with me, the first condition is to use a condom because there are a lot of diseases around here. If he says no, I do not want to but we do it without protection. In that case he has to pay a lot of money. (Age 19, Nacala)*

*It's like this, if he comes and asks for sex with a condom, I charge a reasonable price. If he wants to do it with no condom then the price will be also a little higher. (Age 23, Nacala)*

In some cases, participants reported that use of alcohol or drugs prevented them from enforcing condom use:

*In that case drinking is not very advisable when you are doing this type of work and you may meet a client that does not want to use a condom then as you are drunk you may even accept because you are not focused. (Age 30, Beira)*

*Alcohol is a risk for contracting HIV/AIDS because when we drink, we lose control and end up having sex with no protection. (Age 27, Nacala)*

There were many participants who felt suspicious of clients having “disease” (i.e., STI and HIV infection) that could be transmitted to them, which also contributed to condom use. Many participants also shared the attitude that if a client does not want to use a condom, it may mean he had an infection that he wanted to spread or pass on to them. Participants reported being suspicious of some clients, thinking they might have put holes in their own condoms, and thus preferred asking clients to use the condoms they supply. In some cases, participants reported refusing clients just by physical appearance alone, because of the CSW's perception that the client was sick.

*I always demand using condoms with all my clients because I know that AIDS really exists. So I cannot have sex with no condom. That is the first thing I demand from my clients. If the client does not agree, it is because the client knows that he has a disease for me. I am a prostitute but I must preserve myself because I have children to raise and I know that AIDS exists. Anyone denying the use of condoms has some sort of disease. (Age 29, Nacala)*

Awareness about HIV and a strong sense of protecting the family (not themselves) emerged in the interviews.

*I may have this virus because I do not know what my status is. I use condoms with my boyfriend in order to protect him while with my clients, it depends, there are some that use condoms and others do not. But those I live with, I shall protect them because I do not know what my situation is! (Age 19, Beira)*

Female condoms were mostly unfamiliar to the CSW, with very few of them reporting to have ever used it.

### Knowledge and perceptions about HIV, AID and other STIs

There was a good general understanding among most of the participants of the existence and risk of HIV and AIDS, and other STIs; however some participants were not convinced that HIV was a major health risk. There were also fairly prevalent misconceptions, such as participants reporting that HIV infection can be transmitted by kissing, or sharing cigarettes.

*In practical terms, I do not know [what HIV/AIDS is], I just know the symptoms. In case I feel it I run to my friends. First comes out the discharge, after the discharge the pains, fever, in all a lot of things. This provokes [lets emerge] the STD, then the STD evolves up to AIDS. In order to protect me I first use a condom, avoid kisses, a lot of things, a lot of things that can transmit it. (Age 19, Maputo)*

### Experience with STIs/HIV testing and counselling and other health services

In all three sites some participants reported having used HIV testing and counselling services. Continued problems were reported, however, related to acceptability of services and lack of trust in VCT and other service providers and confidentiality of service provision, with problems being most marked in Nacala, where interviews revealed an especial distrust including the pharmacy were medicines are handed to clients without following confidentiality norms:

*[It would be better] if the consultations were conducted under confidentiality because I know a true story. A friend of mine went to a [HIV testing center] and was told that you have contracted the virus HIV and later he heard about it from a girl friend who heard it from a certain nurse. It created a problem for everyone. I also felt it. So I think [if] I go there they tell me I have the virus HIV and then it gets announced. I will lose my [reputation] in public. I will not be feeling well. If there is a way or a method to treat people while protecting confidentiality so the person alone only knows it [that would improve services]. (Age 19, Nacala)*

*They are good in treating us – it's just that they are not very discrete. You go there and get tested and the results, for example, say you have gonorrhea. Instead of talking to you softly, for example, in the pharmacy while explaining how you should use the medicines, they start shouting so that nearby people hear. (Age 18, Beira)*

In addition to the concerns and distrust on health workers around confidentiality issues, participants refer to having chosen not to be tested for HIV infection in the past, and reported not doing so mostly out of fear of a positive result and the implications a positive test result would have on their mental health and life.

*It is not easy to do the test it is a very difficult decision because a person has to sit and reflect if that is exactly what she wants because from then on a lot of things change. You have to be very strong. I did in 2005 but doing it now I do not feel myself prepared. (Age 27, Maputo)*

When participants felt unwell, particularly if they had symptoms involving the genitals or urinary tract, they visited health centers for screening and treatment; this was reported more often by participants from Beira and Maputo. Having and being treated for an STI was reported as a financial risk, as it requires time off from sex work and thus lost income.

### HIV and STI counseling and testing

All of the 236 CSW participating in the present assessment were offered free HIV counseling and testing and, in Maputo, for syphilis testing as well. As can be seen from Table 3, the mean acceptance of HIV testing was 27% (63/236) in the three cities, with a low of 13% in Nacala. As the results in Table 3 show, half of CSW tested in Maputo, and 55% of those tested in Beira were positive for HIV.

**Table 3:** Results of Voluntary Counseling and Testing (VCT) for HIV and Syphilis, Mozambique, 2007–2008.

	Interviewed	Accepted VCT	HIV Positive	Accepted Syphilis Testing*	Syphilis Test Positive
**Nacala**	85	13% (11/85)	27% (3/11)		
**Beira**	66	33% (22/66)	55% (12/22)		
**Maputo**	85	35% (30/85)	50% (15/30)	35% (30/85)	20% (6/30)
**TOTAL**	236	27% (63/236)	48% (30/63)	35% (30/85)	20% (6/30)

*syphilis testing only offered in Maputo.

In Maputo, the only assessment site where syphilis testing was also offered, all of those presenting for HIV testing also were offered syphilis testing. As can be seen from Table 3, 20% (6/30) of these women tested positive for syphilis.

## Discussion

This assessment has shown that the concept of ‘hard-to-reach’ for CSW may be relative in Mozambique, as once mapping and observation exercises were carried out in the initial stages of the assessments, CSW were reached without excessive difficulties, and were accessible and open to engaging in dialogue and discussion with the interview teams.

The findings reported here demonstrate that CSW mistrust public health personnel and may resist some of the public health and HIV services available, have some misconceptions and gaps in knowledge about HIV, and when they do have correct information it does not always translate into appropriate prevention measures.

The HIV seropositivity rate was 48% among participants who accepted HIV testing in this assessment. This must, however, be considered in light of the low acceptance rate for testing in this group of CSW. It is, for instance, possible that only those who had reason to feel that they were at particular risk were sufficiently motivated to proceed for testing. That would lead to a bias of the result to a deceptively high figure. However it may also be possible that those who did not accept testing were already aware of their status and may be living with HIV. Overall the observed HIV seropositivity rate is consistent with findings in other countries where HIV prevalence among commercial sex workers is higher than in the general population [14]–[16]. The results confirm that in Mozambique, like in other settings, CSW are at very high risk of HIV infection and they highlight the need for targeted HIV interventions for this key population as they have shown to be effective in controlling acquisition and continued spread of HIV [17]–[19]. The low uptake of HIV testing in Nacala may be due both to the prior poor sex worker experience with VCT services (lack of confidentiality) and a less intensive staffing model that was used for providing VCT as part of this assessment in Nacala as compared to other study sites. Syphilis positivity among roughly one-third of Maputo participants was also quite high. Although reliable general-population syphilis statistics are limited, a 2009 “triangulation project” synthesis of data generally reported rates between 2–6% [20]. Another 2002 report from Maputo City looking at legal and illegal abortions found a 4.9% syphilis seropositive rate in the former and 10.9% in the latter group [21].

It is apparent from Table 2 that it is very common for CSW to have children, live with their children, and also often have regular sex partners. Having a family including a husband or cohabiting partner that may or may not be aware of her commercial sex activity has been reported in other international settings [22]–[23]. This may play a key role in HIV and STI prevention, since CSW adhering to condom use with their clients may not use condoms consistently with their regular partners as found in a study in Senegal [16]. In the current study, family and partner patterns were consistent across the three sites, even in Maputo where over 60% list sex work as their main occupation. Also consistent between sites were the stated reasons for entering CSW.

Protective measures for HIV and STI, especially male condom use, were far from uniformly adopted by CSW in the present study. The use of condoms was not consistent, although CSW had knowledge about HIV prevention and the importance of condom use, consistent with findings in similar key populations elsewhere [22], [24]–[26]. Female condoms were barely known by participants and none reported having ever used them, reflecting the lower promotion and almost non-existent distribution system at the time of the study. Though still not widely available, non-governmental organizations with interventions targeted to key populations, especially CSW, are currently promoting and distributing female condoms. Male condoms were reported as widely available, but alcohol intoxication and higher payment per sex act mitigated their use. Oral and anal sex practices were reported but it was not clear whether condoms were used during these practices. There are no reliable data regarding “consumption” of condom commodities nationally, which provides additional opportunity for further investigation within key populations and CSW.

Participants in this study were independent CSW. Whether and to what extent organized sex-work structures exist in the three urban centers is beyond the scope of the present analysis. However, the results we obtained are similar to better-studied independent sex workers in other countries. Perhaps the best studied are the ‘*jineteras*’ of Havana, Cuba. For instance, the study of Cuban sex workers by Kummels [27], motivations were similar to the findings of our study:

*“Three motives incite women to turn to prostitution [in Havana]: the economic necessity of earning a living … their wish to finance or earn starting capital for studies or work in a chosen profession, and finally as a means of pursuing a better, tourist-like lifestyle.”*

In some cases these are individuals who do not need to engage in CSW to cover basic survival needs, but rather to invest in themselves with the goal of gaining a better job, a better life, or simply obtaining the accoutrements of what a better life is perceived to be (eg. paying school tuition or buying desired material items). Often expressed by our interviewees was the desire to purchase designer clothes or other accessories, or to finance other social/leisure activities to keep up with the purchases and trends of friends and other peers. Given the rapidly increasing foreign investment and economic growth in Mozambique, a local form of Mozambican *jinterisma* activity could be comparably increasing.

For the significant number of CSW who identified sex work as their main occupation, it is possible that opportunities to negotiate condom use and decline clients perceived to be “high risk” was more limited. For all CSW, the issues of mistrust and under-utilization of VCT and possibly other HIV and health services may warrant more discussion around what can be done to increase health service utilization among CSW. Health care humanization efforts in Mozambique are underway, with specific attention to key populations in particular pilot settings. Findings should be disseminated and used to inform a national approach. In addition, more robust routine monitoring of services for key populations should be developed, in order to better understand uptake of VCT, HIV care and treatment, STI services, and other health services among CSW and key populations.

Though not comprehensive by virtue of study design, findings from this assessment contribute to strengthening advocacy for programs to be started and expanded for CSW in Mozambique. A strong and committed key population working group based at the National AIDS Council was created and has become the major driving force for influencing national HIV-AIDS policy for key populations. As a result, CSW have been included, for the first time, in the National HIV five year plan as a target group for interventions, and are currently part of the National HIV Response Acceleration Plan that is under development.

## Conclusions

It was possible, using I-RARE methodology, to identify and gain the cooperation of sex workers in three major cities of Mozambique. During this assessment, 236 CSW were interviewed either individually or as part of focus groups. Patterns emerged in the data that indicated poor translation of HIV knowledge into prevention practice as indicated by reported highly uneven and irregular condom use, together with limited use of HIV testing. Irregular condom use emerged as driven mainly out of immediate need of income as higher pay was demanded for sex without condom as well as a result of alcohol and/or drug consumption that lowered CSW capacity to demand for condom use. A small proportion of study participants (27%) opted for subsequent free HIV testing that was offered during the study. Barriers for HIV testing were mainly related to perceived lack of confidentiality and fear of stigma and discrimination. Among those tested, the HIV seropositivity rate was 48%. Considering the burden of the HIV epidemic on commercial sex workers, their relatively limited power to control exposure to HIV and potential position as important nodal points for viral transmission, there is a need for prevention interventions as well as approaches to improve CSW access to STD and HIV services.
